# Multimodal data integration to predict atrial fibrillation

**DOI:** 10.1093/ehjdh/ztae081

**Published:** 2024-11-04

**Authors:** Yuchen Yao, Michael J Zhang, Wendy Wang, Zhong Zhuang, Ruoyu He, Yuekai Ji, Katherine A Knutson, Faye L Norby, Alvaro Alonso, Elsayed Z Soliman, Weihong Tang, James S Pankow, Wei Pan, Lin Yee Chen

**Affiliations:** School of Statistics, College of Liberal Arts, University of Minnesota, 313 Church Street SE, Minneapolis, MN 55455, USA; Division of Biostatistics and Health Data Science, School of Public Health, University of Minnesota, 2221 University Ave SE, Minneapolis, MN 55414, USA; Cardiovascular Division, Department of Medicine, University of Minnesota Medical School, 401 East River Parkway, Minneapolis, MN, USA; Lillehei Heart Institute, University of Minnesota Medical School, 2231 6th Street SE, Minneapolis, MN, USA; Division of Epidemiology and Community Health, School of Public Health, University of Minnesota, 1100 Washington Ave S, Minneapolis, MN 55415, USA; Division of Biostatistics and Health Data Science, School of Public Health, University of Minnesota, 2221 University Ave SE, Minneapolis, MN 55414, USA; School of Statistics, College of Liberal Arts, University of Minnesota, 313 Church Street SE, Minneapolis, MN 55455, USA; Cardiovascular Division, Department of Medicine, University of Minnesota Medical School, 401 East River Parkway, Minneapolis, MN, USA; Lillehei Heart Institute, University of Minnesota Medical School, 2231 6th Street SE, Minneapolis, MN, USA; Division of Biostatistics and Health Data Science, School of Public Health, University of Minnesota, 2221 University Ave SE, Minneapolis, MN 55414, USA; Division of Epidemiology and Community Health, School of Public Health, University of Minnesota, 1100 Washington Ave S, Minneapolis, MN 55415, USA; Department of Epidemiology, Rollins School of Public Health, Emory University, 1518 Clifton Road, Atlanta, GA 30322, USA; Epidemiological Cardiology Research Center, Department of Internal Medicine, Section on Cardiovascular Medicine, Wake Forest School of Medicine, 1 Medical Center Blvd, Winston-Salem, NC 27157, USA; Division of Epidemiology and Community Health, School of Public Health, University of Minnesota, 1100 Washington Ave S, Minneapolis, MN 55415, USA; Division of Epidemiology and Community Health, School of Public Health, University of Minnesota, 1100 Washington Ave S, Minneapolis, MN 55415, USA; Division of Biostatistics and Health Data Science, School of Public Health, University of Minnesota, 2221 University Ave SE, Minneapolis, MN 55414, USA; Cardiovascular Division, Department of Medicine, University of Minnesota Medical School, 401 East River Parkway, Minneapolis, MN, USA; Lillehei Heart Institute, University of Minnesota Medical School, 2231 6th Street SE, Minneapolis, MN, USA

**Keywords:** Atrial fibrillation, Genotype, Proteomics, ECG, Model integration

## Abstract

**Aims:**

Many studies have utilized data sources such as clinical variables, polygenic risk scores, electrocardiogram (ECG), and plasma proteins to predict the risk of atrial fibrillation (AF). However, few studies have integrated all four sources from a single study to comprehensively assess AF prediction.

**Methods and results:**

We included 8374 (Visit 3, 1993–95) and 3730 (Visit 5, 2011–13) participants from the Atherosclerosis Risk in Communities Study to predict incident AF and prevalent (but covert) AF. We constructed a (i) clinical risk score using CHARGE-AF clinical variables, (ii) polygenic risk score using pre-determined weights, (iii) protein risk score using regularized logistic regression, and (iv) ECG risk score from a convolutional neural network. Risk prediction performance was measured using regularized logistic regression. After a median follow-up of 15.1 years, 1910 AF events occurred since Visit 3 and 229 participants had prevalent AF at Visit 5. The area under curve (AUC) improved from 0.660 to 0.752 (95% CI, 0.741–0.763) and from 0.737 to 0.854 (95% CI, 0.828–0.880) after addition of the polygenic risk score to the CHARGE-AF clinical variables for predicting incident and prevalent AF, respectively. Further addition of ECG and protein risk scores improved the AUC to 0.763 (95% CI, 0.753–0.772) and 0.875 (95% CI, 0.851–0.899) for predicting incident and prevalent AF, respectively.

**Conclusion:**

A combination of clinical and polygenic risk scores was the most effective and parsimonious approach to predicting AF. Further addition of an ECG risk score or protein risk score provided only modest incremental improvement for predicting AF.

## Graphical Abstract

**Graphical Abstract:**
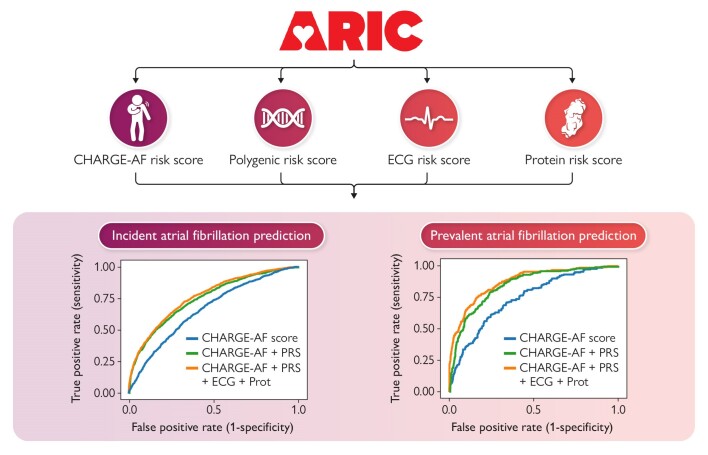

## Introduction

Atrial fibrillation (AF) is the most common type of sustained cardiac arrhythmia, and it is associated with substantial morbidity and mortality.^1^ Therefore, tools to predict the development of AF have substantial public health benefits. Many clinical risk scores (CRSs), such as the Framingham and CHARGE-AF scores, have been developed to predict the risk of AF; however, their predictive performance was moderate.^2–4^

Recent advances in genome-wide association studies have made it possible to construct polygenic risk scores (PRSs) to predict the genetic risk of cardiovascular events, and to combine PRSs with CRSs to improve risk prediction.^5–7^ In a prior AF risk prediction study, the addition of a PRS to the CHARGE-AF CRS resulted in an increase in *C*-index of 0.05.^8^ However, another study showed that the addition of a PRS to a CRS did not result in a *C*-index increase.^9^ Protein biomarkers have also been added to CRSs to improve AF risk prediction: addition of NT-pro-BNP and FGF-23 to a CRS improved the *C*-index by 0.07.^10^ Furthermore, electrocardiogram (ECG)-based models have also been added to CRSs to improve AF risk prediction: addition of a convolution neural network-trained ECG model to the CHARGE-AF score resulted in a *C*-index increase of 0.03.^11^

Despite the large number of studies examining AF risk prediction, few studies have integrated PRS, protein biomarkers, and ECG-based models with CRSs and comprehensively assessed their prediction performance. Therefore, the objective of this study was to develop a PRS, protein biomarker model, and ECG model to individually and collectively add to the CHARGE-AF CRS to improve AF risk prediction. We then comprehensively assessed all model combinations to identify the best combination of models for optimal prediction of AF risk.

## Methods

### Study population and design

**Figure 1:**
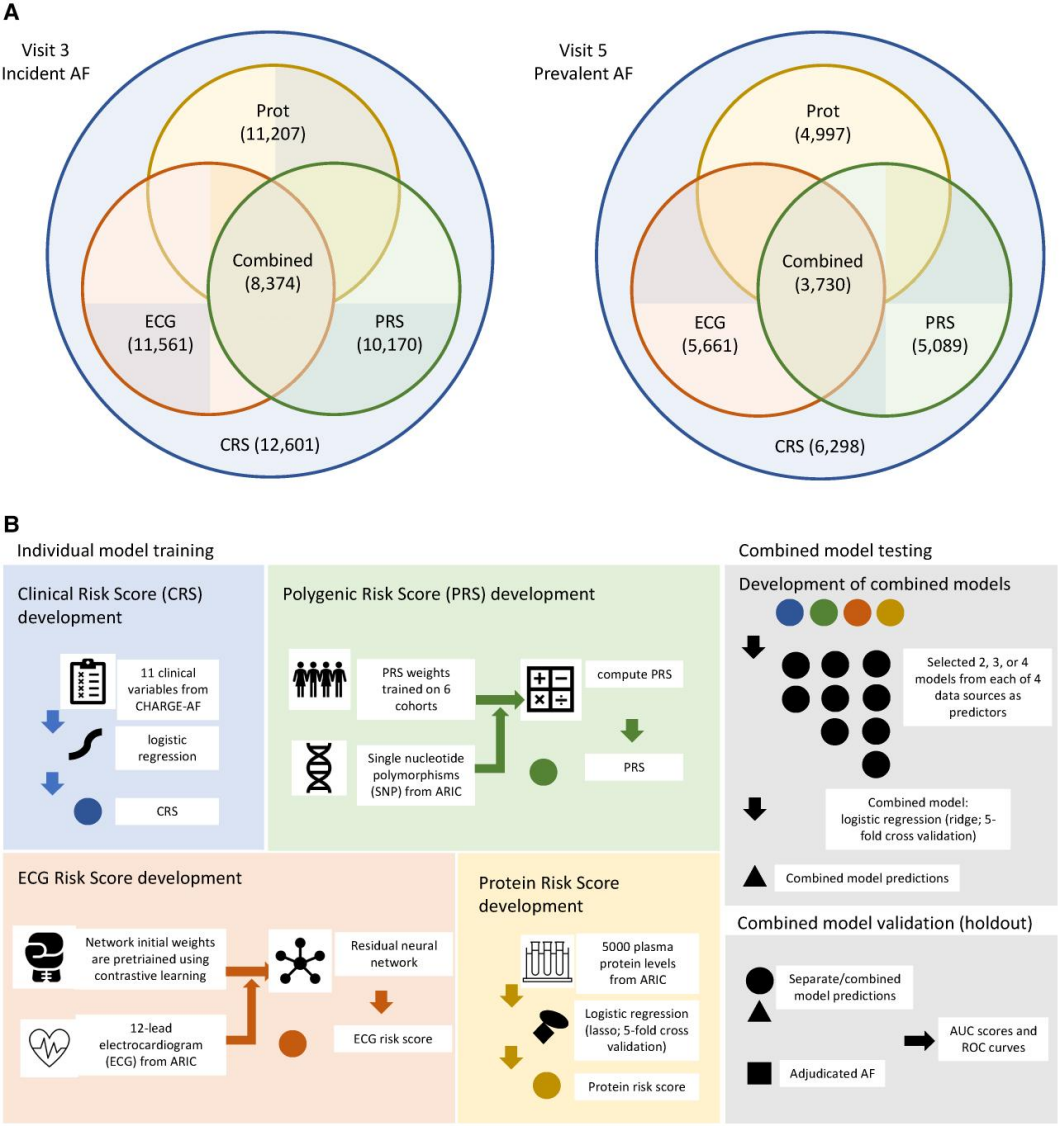

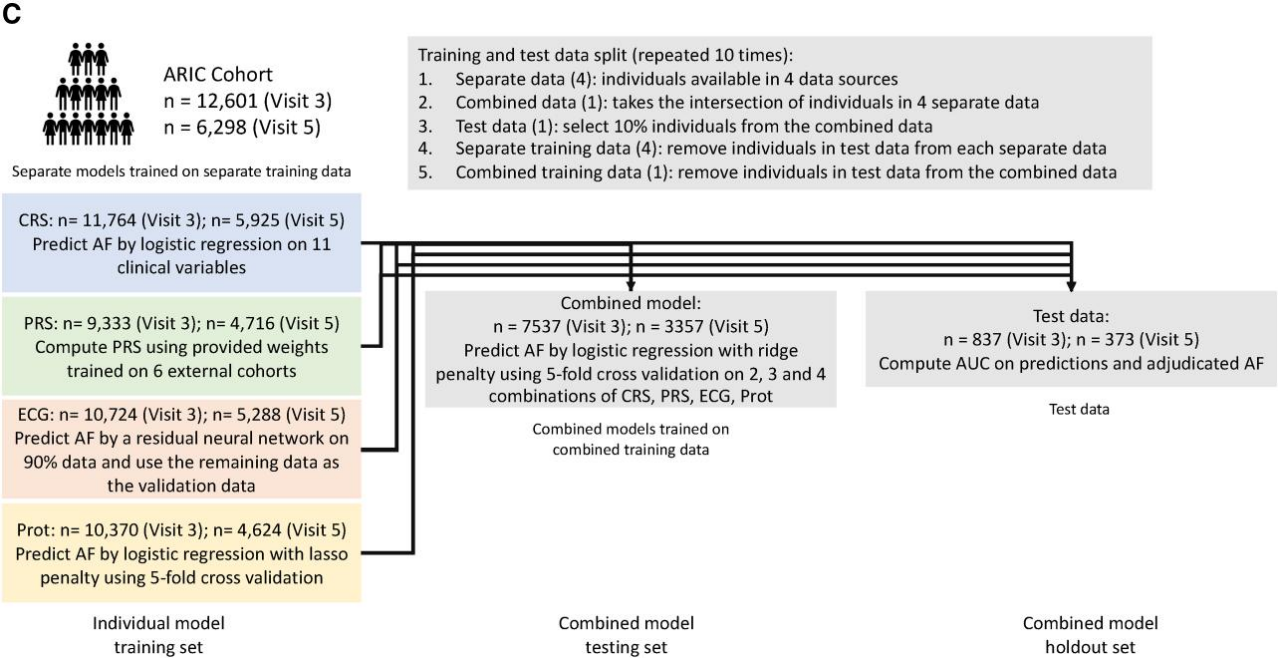
Model development. (*A*) Numbers of participants from different data sources. Diagrams represent the numbers of participants from each of the four data sources for incident AF after Visit 3 (left) and prevalent AF at Visit 5 (right). The combined datasets are intersections among participants in the four data sources, consisting of the test set (consistent across the combined data and four data sources) and the combined training set. For all four data sources, we excluded non-White or Black participants, Black participants in Washington County and Minneapolis centres due to low numbers. We also excluded participants with atrial fibrillation rhythm on the source ECG or poor ECG quality. CRS, clinical risk score; PRS, polygenic risk score; ECG, electrocardiogram model; Prot, protein score. (*B*) Model development diagram. CRS represents the predicted score generated by logistic regression utilizing 11 clinical variables: age, race, height, weight, systolic blood pressure, diastolic blood pressure, current smoking, antihypertensive medication use, diabetes, prevalent heart failure, and prevalent myocardial infarction. PRS was derived with weights provided in a study based on AF GWAS across six cohorts. Combined models integrated information from different combinations of data source. For genotype, ECG, and proteomics, combined models incorporate the risk scores from each data source. For clinical variables, the combined models directly utilized the 11 clinical variables themselves instead of the CRS, proved to produce better prediction performance. (*C*) Diagram of data integration design. Test data remained consistent for comparing both separate and combined models within the same replicate. Sample sizes for incident AF after Visit 3 and prevalent AF at Visit 5 are listed for each dataset. In the case of separate training data for ECG, we additionally partitioned it to network training and validation sets by a ratio of 0.9:0.1. For separate training data regarding proteomics, we employed five-fold cross-validation to choose from 10 values of lasso penalty parameters.

The Atherosclerosis Risk in Communities (ARIC) Study started in 1987 and enrolled 15 792 individuals aged 45–65 years in four communities across the USA: Forsyth County in North Carolina, Jackson in Mississippi, suburbs of Minneapolis in Minnesota, and Washington County in Maryland.^12^ Over the course of more than three decades, ARIC collected an extensive array of clinical data, plasma, and ECGs on its participants across multiple study visits. For this analysis, we included participants who attended the third (Visit 3, 1993–95, *n* = 12 601) and fifth (Visit 5, 2011–13, *n* = 6298) study visits, respectively, who had genotype data, ECG data, and Somalogic-quantified plasma proteomics data. We excluded participants with AF rhythm on their ECGs, with missing clinical covariates, who had low quality proteomics or ECG data, who did not identify as either Black or White, and Black participants from Minneapolis or Washington County due to low participant numbers. A different but overlapping subset of participants had available clinical, genotype, ECG, and proteomics data (*Figure 1A*). In training individual risk prediction models, we included the largest subset of participants with any available clinical, genotype, ECG, or proteomics data. During the combined model training and validation, we included only participants with complete clinical, genotype, ECG, and proteomics data.

The ARIC study obtained approval from the institutional review board at each study site and was conducted in strict adherence to the Declaration of Helsinki, with all participants providing written informed consent.

### Atrial fibrillation outcome ascertainment

Incident AF was defined as AF ascertained after each participant’s Visit 3 date (1993–95) and extending up to the most recent available data (2019).^13,14^ We also evaluated prevalent AF defined as AF occurring on or before each participant’s Visit 5 date (2011–13) but without AF rhythm on their Visit 5 study ECG. We used this definition for prevalent AF to address the common unmet clinical need to identify patients who are at high risk of having AF despite a sinus rhythm ECG (e.g. covert AF in the setting or a recent cardioembolic stroke). Incident and prevalent AF were ascertained using data collected from three sources: 12-lead ECGs collected during study visits, hospital discharge records, and death certificates. The details of the data collection procedure and adjudication of AF have been described previously.^15^

### Clinical covariates

The clinical covariates utilized in this analysis were taken from ARIC Visit 3 and Visit 5, and were based on the CHARGE-AF variables: age, race, cigarette smoking status, height, weight, systolic and diastolic blood pressure, prevalent diabetes, prevalent myocardial infarction, prevalent heart failure, and antihypertensive medication use.^4^ Participants self-reported their race and cigarette smoking status. Blood pressure was measured using an automated sphygmomanometer, with three readings taken and the mean of the last two used for analysis.^16^ Prevalent diabetes was defined as having a fasting glucose level exceeding 126 mg/dL, a non-fasting glucose level surpassing 200 mg/dL, receiving treatment for diabetes mellitus, or having received a self-reported diagnosis of diabetes from a physician.^17,18^ Medication use was documented by study technicians who reviewed the medication bottles brought by study participants during their visits. Estimated glomerular filtration rate was computed using the CKD Epidemiology Collaboration equation, taking into account plasma creatinine and cystatin C measurements.^19^

### Genotype data source

Participant genotypes were acquired through the Affymetrix Genome-Wide Human SNP array 6.0 (HG18 build) at the Broad Institute Center for Genotyping and Analysis, and genotype calling was executed using the Birdseed algorithm as previously described.^20^ Additional genetic variants extending beyond the regions of the genotype array were imputed by leveraging the TOPMED reference panel (freeze 8).^21^ More details for genotyping and imputation procedures can be found in a previously published study.^22^

### Electrocardiogram data source

Electrocardiograms were collected using MAC1200 PC ECG machines (GE Marquette, Milwaukee, WI) using a standardized study protocol with specific instructions on lead placement.^23^ All ECGs were visually inspected by study centre staff to exclude technical errors and suboptimal quality. Following data collection, ECGs were then processed using the 2001 version of the GE Marquette 12-SL program at the EPICORE Center (University of Alberta, Edmonton, Alberta, Canada) and during later phases of the study at the EPICARE Center (Wake Forest University, Winston-Salem, NC). During the data collection phases of Visit 3 and Visit 5, the ECGs were recorded at a rate of 250 and 500 Hz, respectively, capturing data from all 12 leads within a concise 10 s window. Electrocardiogram recordings that were automatically coded as AF by the ECG machine were visually rechecked by a trained cardiologist at each ARIC study centre to confirm the diagnosis. All ECGs coded as AF were excluded from this study because at Visit 3, participants with AF on ECG would be considered a prevalent AF case and would meet analysis exclusion criteria. We also excluded participants with AF rhythm on ECG at Visit 5.

### Proteomics data source

Participant plasma samples from Visit 3 and Visit 5 were stored at −80°C after initial collection and thawed for analysis with the Somalogic 5K aptamer-based proteomics platform in a central laboratory as previously described.^24^ Protein levels were quantified in relative fluorescent units (RFU).

### Statistical analysis

#### Development of the refitted CHARGE-AF clinical risk score

The CHARGE-AF clinical risk score was constructed with a Cox proportional hazards model, designed specifically for the prediction of AF within a 5-year timeframe using pooled data from ARIC, Cardiovascular Health Study (CHS), and Framingham Heart Study.^2,4,25^ Because the CHARGE-AF score was derived using not only ARIC data and calibrated to only a 5-year timeframe, this could potentially bias its performance against the other risk scores used in this paper. Therefore, we refitted the *same* 11 clinical variables of the CHARGE-AF score using only ARIC data and a logistic regression model to develop a *de novo* clinical risk score to predict AF (CRS, *Figure 1B*).

#### Development of the polygenic risk score

A PRS was developed based on pre-determined weights derived from a comparison study of AF polygenic risk and family history for 1 091 491 SNPs (*Figure 1B*).^26^ The weight calculations in that study were conducted using the PRS-CS algorithm,^27^ leveraging data from an AF GWAS^28^ including over one million individuals across six cohorts (HUNT, deCODE, MGI, DIscovEHR, UK Biobank, and the AFGen Consortium), and imputed genotype data on a reference panel sourced from the 1000 Genomes Project.^29^ Due to the difference in the number of available genetic variants between European Americans (EAs) and African Americans (AAs), the PRSs were computed separately for these two distinct ethnic groups in a sensitivity analysis.

#### Development of the electrocardiogram score

An ECG risk score was computed using a neural network architecture derived from a residual 1D convolutional neural network (*Figure 1B*).^30^ The neural network architecture involved four residual convolutional blocks, a global averaging pooling layer and two fully connected layers. The initial weights of the neural network were obtained from an unsupervised contrastive learning study trained on 3.2 million ECGs.^31^ To ensure compatibility with the pretrained model, we transformed the dimensions of the ARIC ECG data, which initially had dimensions of (2500, 12) for Visit 3 and (5000, 12) for Visit 5, into the required format of (4096, 12). Each lead within the ECGs was treated as an individual channel. Within the derivation set designated for ECGs, we further partitioned it into training (90%) and holdout (10%) sample subsets. To address the common issue of overfitting in neural networks, we implemented early stopping using the holdout set, with a patience setting of three.

#### Development of the protein risk score

A protein risk score was developed based on nearly 5000 protein levels (*Figure 1B*). We used logistic regression and incorporated a lasso penalty, characterized by L1 regularization. The inclusion of lasso regularization served the dual purpose of averting overfitting and effectively shrinking the coefficients of less significant protein levels to zero. The process of determining the optimal penalty parameter for lasso involved a five-fold cross-validation procedure considering 10 candidate values. Subsequently, having identified the penalty parameter that yielded the smallest cross-validation error, we proceeded to re-establish a L1 regularized logistic regression model. This model was constructed using all protein levels and applying the chosen penalty parameter across the entire derivation set.

#### Combined model training and risk prediction

During the training and validation phase, for prediction of incident AF after Visit 3 and prevalent AF at Visit 5 in the combined model, we included only participants that had complete genotype, proteomics, and ECG data (*Figure 1C*). Then, we randomly set aside 10% of these participants for the holdout set and used the remaining participants for combined model training (derivation). For the combined model testing, we evaluated the four risk scores on the holdout set.

This entire subset sampling and training framework was repeated 10 times (randomly) to assess the performance of the model across multiple replicates. We developed unified models using logistic regression, which regressed AF outcomes (incident AF or prevalent AF) against the predictors aligned with various combinations of the four data sources (e.g. one, two, three, or four data sources). Logistic regression was utilized instead of Cox regression for incident AF analysis because of better compatibility with the PRS and the neural network-based ECG score and because for relatively short follow-up time and low event rate, the results of logistic and Cox regression are similar.^32^ Considering the modest correlations between predictions among various data sources (see Supplementary material online, *Figure S1*), we added an L2 penalty term. The parameters used in the logistic regression were first standardized to mean 0 and standard deviation 1, a common practice when L2 regularization was used. The optimal choice of the L2 regularization hyperparameter, among 10 candidates, is determined by the best AUC score in five-fold cross-validation. To evaluate the performance of the different data source combinations, we computed area under the ROC curve (AUC) scores on the holdout test set, in which the ROC curve is the curve of the true positive rate vs. the false positive rate. Over the course of 10 replicates, we took the average of the AUC scores obtained from the 10 distinct test sets, ensuring a robust and reliable assessment of model performance. The comparison between the AUC scores from two models was conducted by the paired *t*-test and the DeLong’s test. The paired *t*-test was performed by comparing the differences in AUC scores from two models in 10 replicates. The DeLong’s test was conducted by comparing the two ROC curves generated from the concatenated predictions of two models across 10 replicates. In addition, we also analysed the average of the area under the precision recall curve (PR AUC), which focuses more on handling false positive rates and rare positive cases as compared to the AUC scores. The PR AUC ranged from 0 to 1. A higher PR AUC that was further away from the proportion of positive cases in the data indicated better predictive performance. Finally, for selected models by AUC scores, we applied the Hosmer–Lemeshow (HL) test to each replicate to assess the goodness-of-fit, or calibration, of the data.

## Results

### Study population

*Table 1* displays the baseline characteristics of participants with all four data sources at the time of plasma sample collection at ARIC Visit 3 and Visit 5. Although the sample size at Visit 5 was substantially smaller than Visit 3, the sex and race proportions were comparable between visits. As expected with an older cohort, the proportion of participants with common comorbidities—diabetes, coronary heart disease, heart failure—was substantially higher; antihypertensive medication use was also more prevalent at Visit 5. For this analysis, after a median follow-up of 15.1 years, there were 1910 incident AF cases among 8374 participants at Visit 3 (incidence rate of 12.6 per 1000 person-years). At Visit 5, there were 229 prevalent AF cases among 3730 participants.

**Table 1:** Participant characteristics at the time of plasma collection for protein measurement, Atherosclerosis Risk in Communities study

Variable	ARIC Visit 3	ARIC Visit 5
	*n* = 8374	*n* = 3730
Demographic variables		
Age, years, (SD)	59.98 (5.67)	75.45 (5.05)
Female sex, no. (%)	4541 (54.23)	2141 (57.40)
Male sex, no. (%)	3833 (45.77)	1589 (42.60)
Black race, no. (%)	1660 (19.82)	655 (17.56)
White race, no. (%)	6714 (80.18)	3075 (82.44)
Clinical variables		
Height, cm, (SD)	168.18 (9.42)	165.89 (9.56)
Weight, kg, (SD)	80.70 (17.24)	78.92 (17.24)
Systolic blood pressure, mmHg, (SD)	124.47 (19.14)	129.84 (17.93)
Diastolic blood pressure, mmHg, (SD)	71.79 (10.42)	65.84 (10.53)
Estimated glomerular filtration rate, mL/min/1.73 m^2^, (SD)^a^	89.83 (14.14)	72.05 (17.18)
Prevalent diabetes, no. (%)^b^	1270 (15.17)	1174 (31.47)
Prevalent myocardial infarction, no. (%)^c^	513 (6.13)	429 (11.5)
Prevalent heart failure, no. (%)^d^	67 (0.80)	431 (11.55)
Current smoker, no. (%)	1504 (17.96)	228 (6.11)
Antihypertensive medication use, no. (%)^e^	3116 (37.59)	2779 (74.50)
Prevalent AF, no. (%)	—	229 (6.14)
Black, no. (%)	—	18 (0.48)
White, no. (%)	—	211 (5.66)
Incident AF, no. (%)	1910 (22.81)	—
Black, no. (%)	230 (2.75)	—
Incident AF within 5 years, no. (%)	23 (0.27)	—
Incident AF after 5 years, no. (%)	207 (2.47)	—
White, no. (%)	1680 (20.06)	—
Incident AF within 5 years, no. (%)	207 (2.47)	—
Incident AF after 5 years, no. (%)	1473 (17.59)	—

^a^Estimated glomerular filtration rate was calculated as mL/min/1.73 m^2^ using the CKD Epidemiology Collaboration equation containing plasma creatinine and cystatin C measurements.

^b^Diabetes was defined as having a fasting glucose > 126 mg/dL, a non-fasting glucose > 200 mg/dL, treatment for diabetes mellitus, or a self-reported physician diagnosis of diabetes.

^c^Myocardial infarction was defined as a history of adjudicated myocardial infarction or coronary revascularization procedure.

^d^Heart failure was adjudicated in ARIC by self-reported heart failure medication use within the past two weeks, or ICD-9 code 428.x or ICD-10 code I50 from hospitalization records.

^e^Antihypertensive medications included all medications indicated for hypertension: diuretics, calcium channel blockers, ACE inhibitors, angiotensin II receptor antagonist, adrenergic receptor antagonists, aldosterone receptor antagonists, and alpha-2 adrenergic receptor agonists.

### Prediction of incident atrial fibrillation after ARIC Visit 3

**Figure 2:**
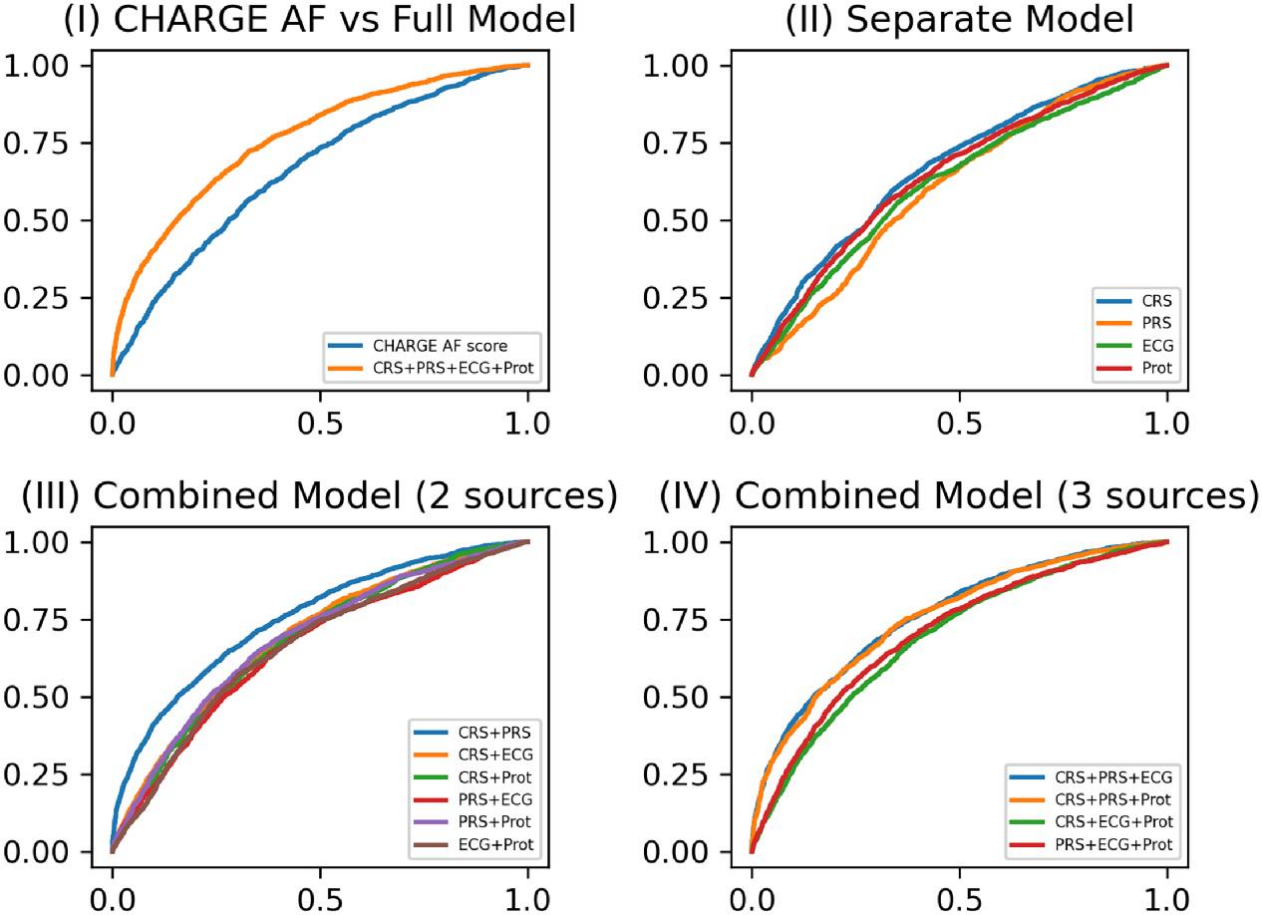
ROC curves for incident atrial fibrillation model prediction performance at Visit 3. (*I*) ROC curves of the base model (CHARGE AF) vs. the full model (CRS + PRS + ECG + Prot); (*II*) ROC curves of four separate models for CRS, PRS, ECG, and protein score, respectively; (*III*) ROC curves of combined models including either two of four data sources (CRS + PRS, CRS + ECG, CRS + Prot, PRS + ECG, PRS + Prot, and ECG + Prot); (*IV*) ROC curves of combined models including either three of four data sources (CRS + PRS + ECG, CRS + PRS + Prot, CRS + ECG + Prot, and PRS + ECG + Prot).

*Table 2* and *Figure 2* present the averaged AUC scores of incident AF prediction after Visit 3 using the various individual and combinations of the four risk scores. The AUC for the base CHARGE-AF risk score was 0.660 (95% CI, 0.648–0.673); refitting this score (CRS) yielded an AUC improvement of 0.008. The AUCs for the individual PRS, ECG, and protein scores were lower than the CRS. After combining two data sources for incident AF risk prediction, the combination of CRS and PRS yielded the largest increase in AUC [0.752 (95% CI, 0.741–0.763)]. After combining three data sources, the combination of CRS, PRS, and ECG scores yielded the largest increase in AUC [0.761 (95% CI, 0.751–0.771)], with the increment of AUC being statistically significant (*P* = 0.001 by the one-sided paired *t*-test and *P* < 0.001 by the one-sided DeLong’s test). The odds ratio for each parameter used in the CRS, PRS, and ECG scores is presented in Supplementary material online, *Table S1*. The PR AUC scores for the combination of CRS and PRS and the combination of CRS, PRS, and ECG scores were 0.518 (95% CI, 0.513–0.524) and 0.525 (95% CI, 0.520–0.531), respectively. The average proportion of incident AF cases in the test sets across 10 replicates was 0.228. The HL tests confirmed that 8/10 and 10/10 replicates passed the goodness-of-fit test for the combination of CRS and PRS and the combination of CRS, PRS, and ECG, respectively; the combination of CRS, PRS, and ECG resulted in a better fit to the observed data. The AUC for the risk score combining all four data sources was even higher [0.763 (95% CI, 0.753–0.772)], but this increase was not statistically significant compared with the combination of CRS, PRS, and ECG scores both by one-sided paired *t*-tests and one-sided DeLong’s tests.

**Table 2:** Incident atrial fibrillation model prediction performance, Atherosclerosis Risk in Communities study (1993–2019)

Data source	AUC (95% CI)	Δ from the base model	Δ from the best model
CHARGE-AF score	0.660 (0.648, 0.673)	—	−0.102
CRS	0.668 (0.657, 0.679)	0.008	−0.094
PRS	0.609 (0.600, 0.617)	−0.052	−0.154
ECG	0.639 (0.617, 0.661)	−0.022	−0.124
Prot	0.645 (0.637, 0.653)	−0.015	−0.118
CRS + PRS	0.752 (0.741, 0.763)	0.091	−0.011
CRS + ECG	0.689 (0.680, 0.699)	0.029	−0.073
CRS + Prot	0.677 (0.668, 0.687)	0.017	−0.085
PRS + ECG	0.671 (0.653, 0.690)	0.011	−0.091
PRS + Prot	0.684 (0.678, 0.691)	0.024	−0.078
ECG + Prot	0.675 (0.663, 0.686)	0.014	−0.088
CRS + PRS + ECG	0.761 (0.751, 0.771)	0.100	−0.002^a,^b^^
CRS + PRS + Prot	0.755 (0.745, 0.766)	0.095	−0.007
CRS + ECG + Prot	0.693 (0.683, 0.703)	0.032	−0.070
PRS + ECG + Prot	0.707 (0.698, 0.715)	0.046	−0.056
CRS + PRS + ECG + Prot	0.763 (0.753, 0.772)	0.102	—

AUC represents averaged AUC scores (and their 95% confidence intervals) of 10 replicates of the indicated data sources. The difference in AUC (Δ) between each model and the base model (CHARGE-AF score) or the best model (with highest averaged AUC score) are also presented. The CRS model is the refitted CHARGE-AF model utilizing the same clinical variables. The model with the largest Δ represents the best model.

AUC, area under curve; CRS, clinical risk score; PRS, polygenic risk score; ECG, electrocardiogram model; Prot, protein score.

^a^Δ is *not* significant in a one-sided paired *t*-test with the best model.

^b^Δ is *not* significant in a one-sided DeLong’s test with the best model.

Since the incidence rates of AF are different in White and Black individuals, and the PRS we used was developed using individuals of predominantly European ancestry, we performed an exploratory race-stratified analysis of the various incident risk prediction scores (see Supplementary material online, *Tables S2* and *S3*). For both White and Black participants, consistent with our main analysis in the whole sample, the CRS and PRS combination yielded the highest AUC among all two data source models, and the CRS, PRS, and ECG combination yielded the highest AUC among all model combinations. Because follow-up time from Visit 3 was ∼25 years, we also performed two separate analyses for incident AF prediction < 5 and >5 years of a participant’s Visit 3 date (see Supplementary material online, *Tables S4* and *S5*). Separating by follow-up time did not change risk prediction results: the CRS and PRS score still yielded the highest AUC for two data source risk prediction; and the CRS, PRS, and ECG combination still yielded the highest overall AUC.

### Prediction of prevalent atrial fibrillation at ARIC Visit 5

**Figure 3:**
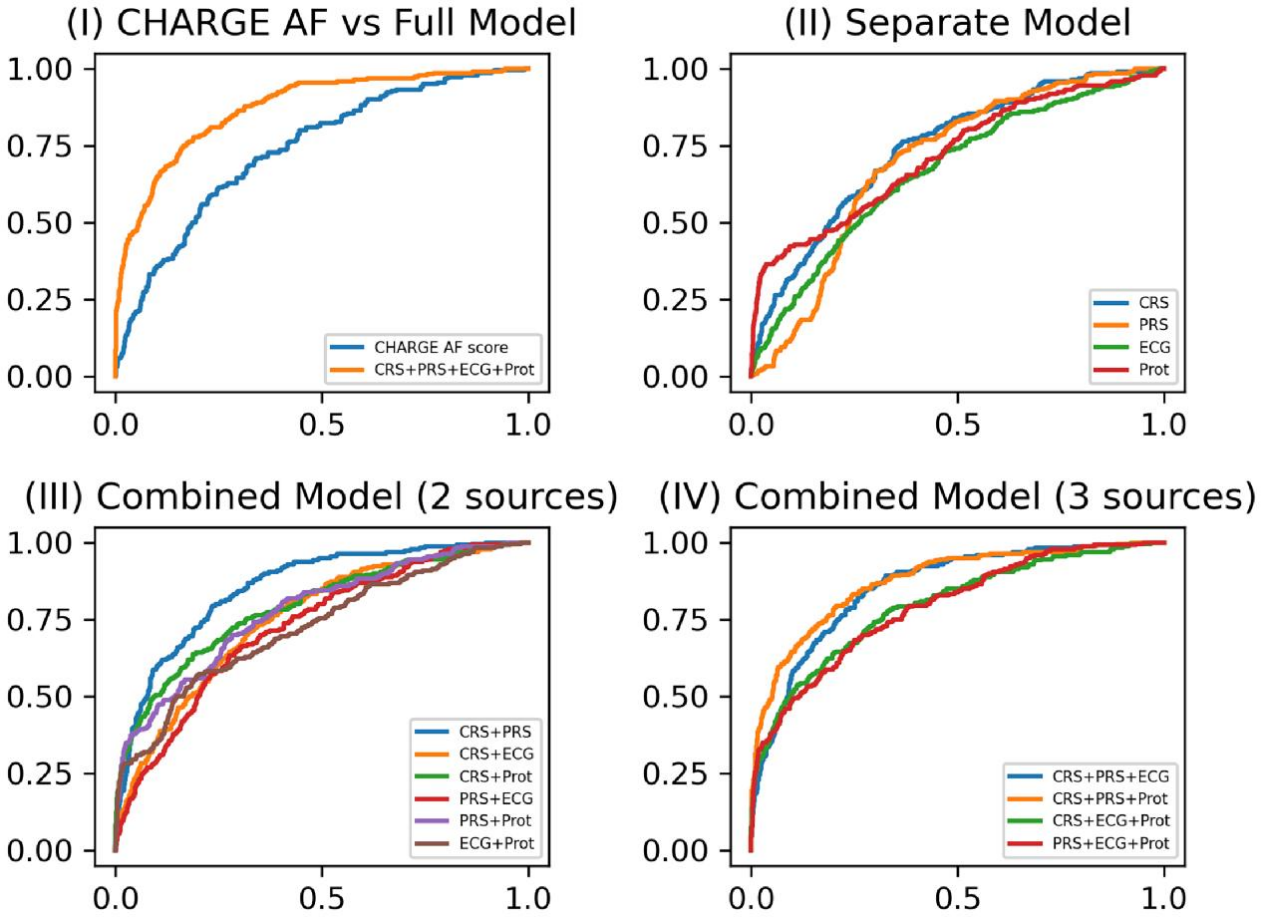
ROC curves for prevalent atrial fibrillation model prediction performance at Visit 5. (*I*) ROC curves of the base model (CHARGE AF) vs. the full model (CRS + PRS + ECG + Pro); (*II*) ROC curves of four separate models for CRS, PRS, ECG, and protein score, respectively; (*III*) ROC curves of combined models including either two of four data sources (CRS + PRS, CRS + ECG, CRS + Prot, PRS + ECG, PRS + Prot, and ECG + Prot); (*IV*) ROC curves of combined models including either three of four data sources (CRS + PRS + ECG, CRS + PRS + Prot, CRS + ECG + Prot, and PRS + ECG + Prot).

*Table 3* and *Figure 3* present the averaged AUC scores of prevalent AF prediction at Visit 5. The AUC for the base CHARGE-AF risk score was 0.737 (95% CI, 0.685–0.789); refitting this score (CRS) yielded an AUC improvement of 0.005. The AUCs for the individual PRS, ECG, and protein scores were lower than the CRS. After combining two data sources for incident AF risk prediction, the combination of CRS and PRS yielded the largest increase in AUC [0.854 (95% CI, 0.828–880)]. After combining three data sources, the combination of CRS, PRS, and protein scores resulted in the largest increase in AUC [0.875 (95% CI, 0.751–0.771)], and the increase of AUC was statistically significant with a *P*-value of 0.005 by the one-sided paired *t*-test and a *P*-value of <0.001 by the one-sided DeLong’s test. The odds ratio for each parameter used in the CRS, PRS, and ECG scores is presented in Supplementary material online, *Table S6*. For the combinations of CRS and PRS scores and the combination of CRS, PRS, and Prot scores, the PR AUC scores were 0.296 (95% CI, 0.277–0.315) and 0.438 (95% CI, 0.416–0.459), respectively. The average proportion of prevalent AF cases in the test sets across 10 replicates was 0.059. Although the addition of the Prot score did not increase the AUC score dramatically, the Prot score greatly improved the PR AUC, which indicated its ability to control false positive rates. The HL tests confirmed that 10/10 and 9/10 replicates passed the goodness-of-fit test for the combination of CRS and PRS and the combination of CRS, PRS, and ECG, respectively. The combination of CRS and PRS resulted in moderately better calibration than the combination of CRS, PRS, and ECG. Adding the ECG score to this model (four data source combination) did not increase the AUC further. When stratified by White race, the same improvements in AUC with data source combinations were observed (see Supplementary material online, *Table S7*). Stratification by Black race could not be completed because there were only 18 prevalent AF cases among 655 Black participants at Visit 5.

**Table 3:** Prevalent atrial fibrillation model diagnostic performance, Atherosclerosis Risk in Communities study Visit 5 (2011–13)

Data source	AUC (95% CI)	Δ from the base model	Δ from the best model
CHARGE-AF score	0.737 (0.685, 0.789)	—	−0.138
CRS	0.742 (0.695, 0.789)	0.005	−0.133
PRS	0.701 (0.682, 0.720)	−0.036	−0.174
ECG	0.671 (0.628, 0.713)	−0.066	−0.204
Prot	0.717 (0.681, 0.753)	−0.020	−0.158
CRS + PRS	0.854 (0.828, 0.880)	0.118	−0.020
CRS + ECG	0.753 (0.715, 0.790)	0.016	−0.122
CRS + Prot	0.793 (0.762, 0.823)	0.056	−0.082
PRS + ECG	0.740 (0.704, 0.775)	0.003	−0.135
PRS + Prot	0.785 (0.757, 0.812)	0.048	−0.090
ECG + Prot	0.747 (0.717, 0.776)	0.010	−0.128
CRS + PRS + ECG	0.855 (0.828, 0.882)	0.119	−0.019
CRS + PRS + Prot	0.875 (0.851, 0.898)	0.138	—
CRS + ECG + Prot	0.797 (0.771, 0.824)	0.061	−0.078
PRS + ECG + Prot	0.797 (0.770, 0.824)	0.060	−0.078
CRS + PRS + ECG + Prot	0.875 (0.851, 0.899)	0.138	—

AUC represents averaged AUC scores (and their 95% confidence intervals) of 10 replicates of the indicated data sources. The difference in AUC (Δ) between each model and the base model (CHARGE-AF score) or the best model (with highest averaged AUC score) are also presented. The CRS model is the refitted CHARGE-AF model utilizing the same clinical variables. The model with the largest Δ represents the best model.

AUC, area under curve; CRS, clinical risk score; PRS, polygenic risk score; ECsG, electrocardiogram model; Prot, protein score.

### Correlations of various risk scores

Next, to investigate the interrelation among risk scores from each data source, thereby explaining their respective contributions to the full four-combination model, we analysed the correlation matrices for incident AF after Visit 3 and prevalent AF at Visit 5 (see Supplementary material online, *Figure S1*). Pearson correlation coefficients between predictions for the test set from these five models were averaged over 10 replicates. For incident AF prediction at Visit 3 and prevalent AF prediction at Visit 5, CRS and protein scores were the primary contributors to the full model (Pearson correlation > 0.4), respectively, whereas PRS exhibited the least influence [0.32 (Visit 3), 0.25 (Visit 5)]. Furthermore, PRS had the lowest correlation with other data sources (all correlations were ≤0.2).

## Discussion

In this analysis of a large Black and White community-based cohort study, the combination of a CRS and PRS was the most effective and parsimonious approach for predicting incident and prevalent AF. For incident AF prediction, addition of an ECG-based risk score provided incremental improvement as compared with the CRS and PRS-based risk score. For prevalent AF prediction, addition of a protein-based risk score provided modest incremental improvement as compared with the CRS and PRS-based risk score, especially in controlling false positive rates.

Many previous studies have developed risk scores based on clinical risk factors, PRS, protein biomarkers, or ECG to predict AF risk. First, Schnabel *et al.*^2^ developed the first AF prediction score in the Framingham Heart Study in 2009 using clinical risk factors and the PR interval on ECG, and achieved an AUC of 0.78. Second, subsequent incorporation of additional cohort studies and clinical risk factors (but not ECG data) into a CHARGE-AF score was unable to improve this initial discrimination performance.^4^ Third, Marston *et al.*^8^ added a PRS to the clinical risk factors and demonstrated an AF prediction AUC improvement of 0.05, but no other data sources were explored. Fourth, Attia *et al.*^33^ used 454 789 ECGs recorded from 126 526 patients to develop a convolutional neural network-derived ECG model to predict AF and achieved an AUC of 0.87. Fifth, Chua *et al.*^34^ used the Olink antibody-based proteomics platform to predict AF and found that the addition of the plasma biomarkers BNP and FGF-23 to the clinical characteristics age, sex, and body mass index improved AF prediction with an AUC improvement of 0.11. Although all of these foregoing studies demonstrated improved AF risk prediction, none of these studies benchmarked their risk scores head-to-head in a single cohort dataset to directly compare AF risk prediction performance.

Therefore, this study advances the field by integrating all four data sources to predict both incident and covert (prevalent) AF, and by directly comparing these data sources using a single cohort dataset to produce insights into the performance of each source vis-à-vis each other. Predicting incident AF is an important clinical need because AF is the most common sustained arrhythmia and it is associated with major adverse cardiac and neurologic morbidity and mortality.^35^ Predicting which patients have covert AF is also an important clinical need because patients with AF may be asymptomatic and therefore may not know they have this clinically significant arrhythmia. Predicting AF risk can also help to personalize and intensify lifestyle modifications to mitigate the burden of AF.^36^ When the various data sources were combined and assessed, the addition of PRS alone to the CRS produced the highest yield increase in both incident and covert (prevalent) AF risk prediction performance. Although the addition of the ECG and protein scores modestly improved AF risk prediction performance, for incident and prevalent AF, respectively, the improvement was likely difficult to justify clinically from a cost perspective. Given that PRS testing costs in the USA are comparable to a single primary care clinic visit and genotype testing (for PRS) needs to only occur once in an individual’s lifetime,^37^ the synergistic combination of CRS and PRS to predict AF appears to be a parsimonious and potentially cost-effective approach. Further research to investigate the cost-effectiveness study of CRS and PRS in predicting AF is warranted.

The predictive performance of the ECG score in our study was lower than the results of the study by Attia *et al*.^33^ Of note, the model used by Attia *et al*. is not publicly available, making direct comparison of the AUC achieved by Attia *et al.* with the AUC achieved in this study impossible. Notwithstanding, the sample size of the study by Attia *et al.* was substantially larger than this study and thus likely in part explains the better model training and subsequent predictive performance. Similarly, the predictive performance of the protein risk score was also modest. It is possible that there was degradation of participant’s plasma proteins during long-term sample storage (at −80°C since 1993). However, a separate validation study for ARIC did not detect widespread protein degradation.^38^

It is important to note that, although sample sizes for training individual models for four data sources were different, missing data were not expected to be a severe issue for the following reasons. First, how the data were missing was largely due to factors like poor ECG quality and sample degradation for SNP and proteomic data, which were expected to be largely random. Second, the missing proportions were small to moderate, ranging from 8% to 20%. Third, in general, it was difficult to accurately impute a high-dimensional ECG or proteomic observation that was missing. Because there were no missing data with covariates, a simple approach was to use the k-nearest neighbour of the covariates to perform imputation: for example, for a given individual A with missing ECG, we could first find their k-nearest neighbours with no missing ECGs (i.e. k individuals with their standardized covariate values closest in Euclidean distance to those of A), then we randomly selected one of the k individuals and used the selected individual’s ECG as the ECG for individual A. We compared the predictive performance for AF with the ECG and protein risk scores, respectively, with or without imputation, using k = 10 in the first replicate of our original experiment. We repeated the process five times (i.e. with multiple imputation) and reported the median AUC scores for incident and prevalent AF (see Supplementary material online, *Table S8*). There were no significant differences, by DeLong’s test, between the AUCs obtained with the data before and after imputation. We also noted that the PRS reported here was computed based on fixed weights from an external dataset; therefore, training on imputed or non-imputed data would not affect model performance. Overall, these findings lent further support for our current analysis, but future validation with external or independent data is warranted.

### Limitations

First, the sample size of our study was moderate compared to many other AF prediction studies. However, our dataset is currently the *largest* one to integrate data from four sources, which allowed us to directly compare risk score performance *within* the same cohort. Second, some participants in our Visit 5 prevalent AF analysis may have experienced symptomatic AF that resolved by the time of their Visit 5 study ECG (and therefore did not demonstrate AF on ECG). Nevertheless, because all clinical variables, plasma protein, and ECG data were collected on the study visit date without the presence of AF rhythm, our source data represented bona fide prevalent AF. Third, we treated censored individuals without AF as controls for the incident AF analysis, which might introduce misclassification bias. However, given the relatively small numbers of such individuals, such a bias was expected to be minimal: at Visit 3, among 8374 participants, 345 were censored (without AF diagnosed) within 5 years. Fourth, although survival analysis using Cox regression can properly deal with censoring, applying Cox regression to neural networks was problematic and therefore not performed. More importantly, our numerical results consistently demonstrated a close performance between the Cox model-based CHARGE-AF and our logistic model-based CRS. Fifth, the CHARGE-AF model was based on a 5-year Cox regression model, but our primary incident AF analysis extended up to 25 years. However, we performed an additional analysis limiting to follow-up to 5 years (see Supplementary material online, *Table S4*) and our results were close (CRS + PRS was the best combination of scores and CRS + PRS + ECG did not perform significantly worse than CRS + PRS). Fifth, the PRS used in this study was based on individuals of primarily European ancestry, thus explaining the lower AF risk prediction performance in Black participants (see Supplementary material online, *Table S3*). However, the objective of this study was to compare different risk scores and not to specifically develop the best PRS. Future studies that develop multi-ancestry or ancestry-specific PRSs are warranted.

## Conclusion

A combination of clinical and PRSs was the most effective and parsimonious approach to predicting AF. Further addition of an ECG risk score or protein risk score provided only modest incremental improvement for predicting AF.

## Supplementary Material

ztae081_Supplementary_Data

## Data Availability

The data that support the findings of this study are not openly available due to reasons of sensitivity and are available from the corresponding author upon reasonable request. Data are located in controlled access data storage at the ARIC Data Coordinating Center at University of North Carolina, Chapel Hill. All software used in this study are publicly available: Python v3, R v4. The code used in this study can be made available from the corresponding author upon reasonable request.
