# Supplementary material for: Multimodal data integration to predict atrial fibrillation
Source: Eur Heart J Digit Health. 2024 Nov 4;6(1):126–36. doi: 10.1093/ehjdh/ztae081 (PMC11750194; doi:10.1093/ehjdh/ztae081)
Supplement: ztae081_Supplementary_Data [file ztae081_supplementary_data.docx]

**Supplemental Material**

**Supplemental Table 1. Incident Atrial Fibrillation Model Odds Ratios Among White and Black Participants, Atherosclerosis Risk in Communities Study Visit 3 (1993-2019)**

| Variables | CRS + PRS  OR (95%CI) | CRS + PRS + ECG  OR (95%CI) |
| --- | --- | --- |
| Age (SD) | 1.41 (1.40,1.43) | 1.33 (1.30,1.36) |
| Height (SD) | 1.14 (1.13,1.15) | 1.10 (1.09,1.12) |
| Weight (SD) | 1.32 (1.31,1.32) | 1.27 (1.24,1.29) |
| Systolic BP (SD) | 1.21 (1.20,1.22) | 1.16 (1.14,1.19) |
| Diastolic BP (SD) | 0.87 (0.86,0.87) | 0.88 (0.87,0.89) |
| Smoking (Yes) | 1.23 (1.22,1.24) | 1.18 (1.16,1.20) |
| Anti-hypertensive medication use (Yes) | 1.31 (1.29,1.33) | 1.25 (1.21,1.28) |
| Diabetes (Yes) | 0.95 (0.92,0.97) | 0.92 (0.90,0.94) |
| Heart failure (Yes) | 1.18 (1.12,1.25) | 1.01 (0.92,1.11) |
| Myocardial infarction (Yes) | 1.29 (1.27,1.31) | 1.16 (1.08,1.24) |
| Polygenic risk score (SD) | 2.74 (2.68,2.81) | 2.65 (2.58,2.71) |
| ECG risk score (SD) | --- | 1.36 (1.23,1.51) |

OR (odds ratio) represents the averaged odds ratios (and their 95% confidence intervals) from 10 replicates of the parameters used in the CRS + PRS or CRS + PRS + ECG model. All parameters were standardized (mean=0 and standard deviation=1) before fitting a logistic regression. Odds ratios for Race was omitted due to its modest correlation with PRS, leading to difficulty in interpretation. The values in columns 2-3 can be interpreted as the odds of incident AF per one standard deviation increase of continues variables or per switch from No to Yes for binary variables.

Abbreviations: CRS, clinical risk score; PRS, polygenic risk score; ECG, electrocardiogram model.

**Supplemental Table 2. Incident Atrial Fibrillation Model Prediction Performance Among White Participants, Atherosclerosis Risk in Communities Study Visit 3 (1993-2019)**

| Data source | AUC (95%CI) | Δ from the base model | Δ from the best model |
| --- | --- | --- | --- |
| CHARGE-AF score | 0.662 (0.644,0.679) | --- | -0.087 |
| CRS | 0.662 (0.645,0.679) | 0.001 | -0.086 |
| PRS | 0.691 (0.681,0.702) | 0.030 | -0.057 |
| ECG | 0.596 (0.558,0.633) | -0.066 | -0.153 |
| Prot | 0.631 (0.615,0.648) | -0.030 | -0.117 |
| CRS+PRS | 0.745 (0.732,0.758) | 0.083 | -0.004^a,b^ |
| CRS+ECG | 0.671 (0.657,0.685) | 0.010 | -0.077 |
| CRS+Prot | 0.668 (0.650,0.686) | 0.007 | -0.081 |
| PRS+ECG | 0.711 (0.691,0.730) | 0.049 | -0.038 |
| PRS+Prot | 0.718 (0.704,0.731) | 0.056 | -0.031 |
| ECG+Prot | 0.650 (0.631,0.669) | -0.012 | -0.099 |
| CRS+PRS+ECG | 0.749 (0.735,0.762) | 0.087 | --- |
| CRS+PRS+Prot | 0.745 (0.732,0.759) | 0.084 | -0.003^a,b^ |
| CRS+ECG+Prot | 0.677 (0.662,0.692) | 0.016 | -0.071 |
| PRS+ECG+Prot | 0.727 (0.710,0.744) | 0.065 | -0.022 |
| CRS+PRS+ECG+Prot | 0.748 (0.734,0.763) | 0.087 | -0.000^a,b^ |

AUC represents averaged AUC scores (and their 95% confidence intervals) of 10 replicates of the indicated data sources. The difference in AUC (Δ) between each model and the base model (CHARGE-AF score) or the best model (with highest averaged AUC score) are also presented. The CRS model is the refitted CHARGE-AF model utilizing the same clinical variables. The model with the largest Δ represents the best model.

^a^ Δ is *NOT* significant in a one-sided paired t-test with the best model.

^b^ Δ is *NOT* significant in a one-sided DeLong’s test with the best model.

Abbreviations: AUC, area under curve; CRS, clinical risk score; PRS, polygenic risk score; ECG, electrocardiogram model; Prot, protein score.

**Supplemental Table 3. Incident Atrial Fibrillation Model Prediction Performance Among Black Participants, Atherosclerosis Risk in Communities Study Visit 3 (1993-2019)**

| Data source | AUC (95%CI) | Δ from the base model | Δ from the best model |
| --- | --- | --- | --- |
| CHARGE-AF score | 0.608 (0.572,0.643) | --- | -0.052 |
| CRS | 0.602 (0.569,0.636) | -0.005 | -0.057 |
| PRS | 0.632 (0.594,0.670) | 0.024 | -0.028 |
| ECG | 0.540 (0.492,0.589) | -0.067 | -0.119 |
| Prot | 0.539 (0.511,0.568) | -0.068 | -0.120 |
| CRS+PRS | 0.658 (0.631,0.684) | 0.050 | -0.002^a,b^ |
| CRS+ECG | 0.607 (0.581,0.633) | -0.000 | -0.053 |
| CRS+Prot | 0.576 (0.547,0.605) | -0.032 | -0.084 |
| PRS+ECG | 0.642 (0.606,0.678) | 0.034 | -0.018 ^b^ |
| PRS+Prot | 0.617 (0.584,0.650) | 0.010 | -0.042 |
| ECG+Prot | 0.554 (0.532,0.576) | -0.053 | -0.106 |
| CRS+PRS+ECG | 0.660 (0.635,0.685) | 0.052 | --- |
| CRS+PRS+Prot | 0.635 (0.608,0.662) | 0.027 | -0.025 |
| CRS+ECG+Prot | 0.580 (0.554,0.606) | -0.028 | -0.080 |
| PRS+ECG+Prot | 0.623 (0.590,0.656) | 0.015 | -0.037 |
| CRS+PRS+ECG+Prot | 0.641 (0.616,0.666) | 0.033 | -0.019 ^b^ |

AUC represents averaged AUC scores (and their 95% confidence intervals) of 10 replicates of the indicated data sources. The difference in AUC (Δ) between each model and the base model (CHARGE-AF score) or the best model (with highest averaged AUC score) are also presented. The CRS model is the refitted CHARGE-AF model utilizing the same clinical variables. The model with the largest Δ represents the best model.

^a^ Δ is *NOT* significant in a one-sided paired t-test with the best model.

^b^ Δ is *NOT* significant in a one-sided DeLong’s test with the best model.

Abbreviations: AUC, area under curve; CRS, clinical risk score; PRS, polygenic risk score; ECG, electrocardiogram model; Prot, protein score.

**Supplemental Table 4. Incident Atrial Fibrillation (< 5 years) Model Prediction Performance Among White and Black Participants, Atherosclerosis Risk in Communities Study Visit 3 (1993-2019)**

| Data source | AUC (95%CI) | Δ from the base model | Δ from the best model |
| --- | --- | --- | --- |
| CHARGE-AF score | 0.753 (0.726,0.779) | --- | -0.095 |
| CRS | 0.750 (0.725,0.775) | -0.002 | -0.098 |
| PRS | 0.677 (0.662,0.692) | -0.075 | -0.171 |
| ECG | 0.715 (0.690,0.739) | -0.038 | -0.133 |
| Prot | 0.662 (0.627,0.698) | -0.090 | -0.186 |
| CRS+PRS | 0.848 (0.832,0.864) | 0.095 | --- |
| CRS+ECG | 0.767 (0.746,0.788) | 0.015 | -0.080 |
| CRS+Prot | 0.739 (0.709,0.769) | -0.014 | -0.109 |
| PRS+ECG | 0.754 (0.732,0.777) | 0.002 | -0.094 |
| PRS+Prot | 0.746 (0.715,0.776) | -0.007 | -0.102 |
| ECG+Prot | 0.731 (0.708,0.753) | -0.022 | -0.117 |
| CRS+PRS+ECG | 0.845 (0.833,0.856) | 0.092 | -0.003^a,b^ |
| CRS+PRS+Prot | 0.843 (0.822,0.864) | 0.091 | -0.005^a,b^ |
| CRS+ECG+Prot | 0.762 (0.742,0.783) | 0.010 | -0.085 |
| PRS+ECG+Prot | 0.780 (0.762,0.797) | 0.027 | -0.068 |
| CRS+PRS+ECG+Prot | 0.844 (0.831,0.858) | 0.092 | -0.004^a,b^ |

AUC represents averaged AUC scores (and their 95% confidence intervals) of 10 replicates of the indicated data sources. The difference in AUC (Δ) between each model and the base model (CHARGE-AF score) or the best model (with highest averaged AUC score) are also presented. The CRS model is the refitted CHARGE-AF model utilizing the same clinical variables. The model with the largest Δ represents the best model.

^a^ Δ is *NOT* significant in a one-sided paired t-test with the best model.

^b^ Δ is *NOT* significant in a one-sided DeLong’s test with the best model.

Abbreviations: AUC, area under curve; CRS, clinical risk score; PRS, polygenic risk score; ECG, electrocardiogram model; Prot, protein score.

**Supplemental Table 5. Incident Atrial Fibrillation (> 5 years) Model Prediction Performance Among White and Black Participants, Atherosclerosis Risk in Communities Study Visit 3 (1993-2019)**

| Data source | AUC (95%CI) | Δ from the base model | Δ from the best model |
| --- | --- | --- | --- |
| CHARGE-AF score | 0.632 (0.620,0.644) | --- | -0.085 |
| CRS | 0.640 (0.631,0.649) | 0.008 | -0.076 |
| PRS | 0.591 (0.581,0.601) | -0.041 | -0.125 |
| ECG | 0.589 (0.553,0.626) | -0.043 | -0.127 |
| Prot | 0.618 (0.609,0.628) | -0.013 | -0.098 |
| CRS+PRS | 0.710 (0.699,0.721) | 0.078 | -0.006 |
| CRS+ECG | 0.654 (0.640,0.667) | 0.022 | -0.063 |
| CRS+Prot | 0.643 (0.633,0.654) | 0.011 | -0.073 |
| PRS+ECG | 0.630 (0.609,0.652) | -0.001 | -0.086 |
| PRS+Prot | 0.653 (0.645,0.661) | 0.021 | -0.063 |
| ECG+Prot | 0.639 (0.622,0.656) | 0.007 | -0.077 |
| CRS+PRS+ECG | 0.716 (0.706,0.726) | 0.085 | --- |
| CRS+PRS+Prot | 0.709 (0.700,0.717) | 0.077 | -0.007 |
| CRS+ECG+Prot | 0.654 (0.641,0.667) | 0.022 | -0.062 |
| PRS+ECG+Prot | 0.670 (0.658,0.683) | 0.039 | -0.046 |
| CRS+PRS+ECG+Prot | 0.715 (0.706,0.724) | 0.083 | -0.001^a,b^ |

AUC represents averaged AUC scores (and their 95% confidence intervals) of 10 replicates of the indicated data sources. The difference in AUC (Δ) between each model and the base model (CHARGE-AF score) or the best model (with highest averaged AUC score) are also presented. The CRS model is the refitted CHARGE-AF model utilizing the same clinical variables. The model with the largest Δ represents the best model.

^a^ Δ is *NOT* significant in a one-sided paired t-test with the best model.

^b^ Δ is *NOT* significant in a one-sided DeLong’s test with the best model.

Abbreviations: AUC, area under curve; CRS, clinical risk score; PRS, polygenic risk score; ECG, electrocardiogram model; Prot, protein score.

**Supplemental Table 6. Prevalent Atrial Fibrillation Model Odds Ratios, Atherosclerosis Risk in Communities Study Visit 5 (2011-2013)**

| Variables | CRS + PRS  OR (95%CI) | CRS + PRS + Prot  OR (95%CI) |
| --- | --- | --- |
| Age (SD) | 1.67 (1.64,1.70) | 1.56 (1.53,1.58) |
| Height (SD) | 1.25 (1.23,1.27) | 1.18 (1.16,1.20) |
| Weight (SD) | 1.10 (1.08,1.12) | 1.09 (1.07,1.12) |
| Systolic BP (SD) | 1.09 (1.07,1.11) | 1.16 (1.12,1.20) |
| Diastolic BP (SD) | 0.91 (0.90,0.93) | 0.90 (0.87,0.92) |
| Smoking (Yes) | 0.89 (0.81,0.97) | 1.08 (1.00,1.17) |
| Anti-hypertensive medication use (Yes) | 1.74 (1.67,1.81) | 1.76 (1.67,1.86) |
| Diabetes (Yes) | 0.93 (0.90,0.95) | 1.00 (0.97,1.03) |
| Heart failure (Yes) | 3.61 (3.51,3.71) | 2.79 (2.68,2.91) |
| Myocardial infarction (Yes) | 1.67 (1.60,1.74) | 1.62 (1.54,1.71) |
| Polygenic risk score (SD) | 4.40 (4.29,4.51) | 4.47 (4.36,4.59) |
| Protein risk score (SD) | --- | 1.86 (1.83,1.90) |

OR represents the averaged odds ratios (and their 95% confidence intervals) from 10 replicates of the parameters used in the CRS + PRS or CRS + PRS + Prot model. All parameters were standardized (mean=0 and standard deviation=1) before fitting a logistic regression. Odds ratios for Race was omitted due to its modest correlation with PRS, leading to difficulty in interpretation. The values in columns 2-3 can be interpreted as the odds of prevalent AF per one standard deviation increase of continues variables or per switch from No to Yes for binary variables.

Abbreviations: CRS, clinical risk score; PRS, polygenic risk score; Prot, protein score.

**Supplemental Table 7. Prevalent Atrial Fibrillation Model Prediction Performance Among White Participants, Atherosclerosis Risk in Communities Study Visit 5 (2011-2013)**

| Data source | AUC (95%CI) | Δ from the base model | Δ from the best model |
| --- | --- | --- | --- |
| CHARGE-AF score | 0.736 (0.713,0.759) | --- | -0.127 |
| CRS | 0.730 (0.701,0.758) | -0.006 | -0.133 |
| PRS | 0.778 (0.744,0.812) | 0.042 | -0.085 |
| ECG | 0.654 (0.565,0.742) | -0.082 | -0.209 |
| Prot | 0.710 (0.672,0.748) | -0.026 | -0.153 |
| CRS+PRS | 0.847 (0.827,0.867) | 0.111 | -0.016 |
| CRS+ECG | 0.756 (0.729,0.784) | 0.020 | -0.107 |
| CRS+Prot | 0.778 (0.752,0.804) | 0.042 | -0.085 |
| PRS+ECG | 0.808 (0.785,0.831) | 0.072 | -0.055 |
| PRS+Prot | 0.827 (0.807,0.847) | 0.091 | -0.036 |
| ECG+Prot | 0.753 (0.712,0.795) | 0.017 | -0.110 |
| CRS+PRS+ECG | 0.850 (0.830,0.870) | 0.114 | -0.013 |
| CRS+PRS+Prot | 0.862 (0.846,0.877) | 0.126 | -0.001^a,b^ |
| CRS+ECG+Prot | 0.789 (0.764,0.814) | 0.053 | -0.074 |
| PRS+ECG+Prot | 0.839 (0.821,0.857) | 0.103 | -0.024 |
| CRS+PRS+ECG+Prot | 0.863 (0.848,0.878) | 0.127 | --- |

AUC represents averaged AUC scores (and their 95% confidence intervals) of 10 replicates of the indicated data sources. The difference in AUC (Δ) between each model and the base model (CHARGE-AF score) or the best model (with highest averaged AUC score) are also presented. The CRS model is the refitted CHARGE-AF model utilizing the same clinical variables. The model with the largest Δ represents the best model.

^a^ Δ is *NOT* significant in a one-sided paired t-test with the best model.

^b^ Δ is *NOT* significant in a one-sided DeLong’s test with the best model.

Abbreviations: AUC, area under curve; CRS, clinical risk score; PRS, polygenic risk score; ECG, electrocardiogram model; Prot, protein score.

**Supplemental Table 8. Comparison of Atrial Fibrillation Model Prediction Performance With or Without Imputation Among White and Black Participants**

| Data source | AUC of the  original model | AUC of the new model  Trained with imputation | *P-value of Δ  between AUCs |
| --- | --- | --- | --- |
| Incident AF (Visit 3) |  |  |  |
| ECG | 0.608 | 0.563 | 0.114 |
| Prot | 0.637 | 0.638 | 0.747 |
| Prevalent AF (Visit 5) |  |  |  |
| ECG | 0.692 | 0.671 | 0.734 |
| Prot | 0.690 | 0.719 | 0.174 |

AUC represents the AUC score of the first replicate of the ECG data or proteomics. *The p-values of two-sided DeLong’s tests between AUC scores in the two models are also presented.

Abbreviations: AUC, area under curve; ECG, electrocardiogram model; Prot, protein score.

**Supplemental Figure 1. Correlation Coefficients Between the Predictions from Separate and the Full Models for Incident AF After Visit 3 or Prevalent AF at Visit 5**

Each value denotes the correlation between predictions from either two of four separate models (CRS, PRS, ECG, Prot) and the full model (CRS + PRS + ECG + Prot) for incident AF after Visit 3 or prevalent AF at Visit 5. For example, in the left graph for incident AF after Visit 3, the value of 0.60 in the lower-left box represents a correlation of 0.60 between the predictions from the separate model CRS and the predictions from the full model.

Abbreviations: CRS, clinical risk score; PRS, polygenic risk score; ECG, electrocardiogram model; Prot, protein score.
